# Neurocognitive Correlates of Apathy and Anxiety in Parkinson's Disease

**DOI:** 10.1155/2012/793076

**Published:** 2011-12-12

**Authors:** Yelena Bogdanova, Alice Cronin-Golomb

**Affiliations:** ^1^Department of Psychology, Boston University, Boston, MA 02215, USA; ^2^Psychology Research (151-A), VA Boston Healthcare System, 150 South Huntington Street, Boston, MA 02130, USA; ^3^Department of Psychiatry, Boston University School of Medicine, Boston, MA 02118, USA; ^4^Department of Psychiatry, Harvard Medical School, Boston, MA 02115, USA

## Abstract

Parkinson's disease (PD) is associated with various nonmotor symptoms including neuropsychiatric and cognitive dysfunction. We examined the relation between apathy, anxiety, side of onset of motor symptoms, and cognition in PD. We hypothesized that PD patients would show different neuropsychiatric and neurocognitive profiles depending on the side of onset. 22 nondemented PD patients (11 right-side onset (RPD) with predominant left-hemisphere pathology, and 11 LPD) and 22 matched healthy controls (NC) were administered rating scales assessing apathy and anxiety, and a series of neuropsychological tests. PD patients showed a higher anxiety level than NC. There was a significant association between apathy, anxiety, and disease duration. In LPD, apathy but not anxiety was associated with performance on nonverbally mediated executive function and visuospatial measures, whereas, in RPD, anxiety but not apathy correlated with performance on verbally mediated tasks. Our findings demonstrated a differential association of apathy and anxiety to cognition in PD.

## 1. Introduction

Parkinson's disease (PD) is a progressive neurodegenerative disorder characterized by the loss of nigrostriatal and mesocortical dopaminergic projections from the brain stem and limbic cortex to the basal ganglia and neocortex. The basal ganglia and related structures are critical structures in parallel frontal subcortical circuits involved in regulation of cognition, emotions, and behavioral activation [1–3]. While primarily characterized as a movement disorder, PD is associated with various nonmotor symptoms [4–6], including cognitive dysfunction [7–10] and neuropsychiatric symptoms, such as apathy [11–17] and anxiety [18–20]. Recent neuroimaging and neuropsychological findings raise the question of the role of apathy and anxiety in PD-related cognitive dysfunction, as these neuropsychiatric conditions reflect dysfunction in brain areas involved in PD.

Typically, early motor signs of PD start on one side of the body, and side of onset remains a significant clinical and neuropathological factor in both clinical management and the study of PD. PD patients with symptoms starting on the right side of the body (RPD) have greater inferred left hemisphere pathology and those with left-side onset (LPD) have greater inferred right hemisphere pathology [21]. The motor symptoms of PD are associated with asymmetrical depletion of dopamine in the substantia nigra of the midbrain across the range of disease severity. These changes in the substantia nigra lead to asymmetrical dysregulation of the striatum, which may in turn lead to further asymmetrical dysfunction of neural circuits including the basal ganglia and cortical projection areas (reviewed in [8]). This neuropathological asymmetry remains evident even with PD progression [22], and initial motor asymmetry predicts a range of motor and neuropsychiatric deficits in PD patients [21, 23]. Specific cognitive problems are also related to side of initial motor onset: LPD versus RPD [8].

Cognitive impairments to a various degree have been documented in many patients with PD. The most common cognitive domains affected are attention and executive function (planning, problem solving, verbal fluency) and visuospatial function. While language abilities remain generally intact, mild naming deficits may be present in some patients [24]. Previous work demonstrated that the degree of impairment and specificity of cognitive domain affected by the disease is mediated by the side of initial motor onset, which is presumably predetermined by asymmetrical dopamine depletion in PD (reviewed in [8]). Thus, cognitive domains predominantly subserved by the left hemisphere, such as verbally mediated tasks of executive function and verbal memory would be more affected in RPD than LPD, and vice versa. For instance, RPD patients showed poorer verbal memory performance than do those with LPD, who were more impaired on visual memory [7].

Apathy, a reduction in self-initiated cognitive, emotional, and behavioral activity, is a common clinical feature of prefrontal and basal ganglia lesions or dysfunctions [25]. Apathy was documented following direct damage to the prefrontal cortex [26–28]. It is also a common feature of frontostriatal diseases, such as PD [11–14], Huntington's disease [29–31], and HIV [32–37]. Apathy in PD is attributed to nigrostriatal dopaminergic loss in basal ganglia [38] and is considered one of the core features of PD [17], occurring in up to 70% of PD patients [39]. While apathy symptoms may overlap with those of depression, the two conditions have been shown to be reliably differentiated in patient samples [16, 40]. Apathy was related to cognitive dysfunction in PD and other frontostriatal disorders. Specifically, apathy was associated with more severe cognitive dysfunction, specifically executive dysfunction in PD [13, 14, 41]. Studies of HIV have documented associations between the presence of apathy and poor performance on measures of executive function, suggesting that apathy and HIV-related cognitive dysfunction may share common neurophysiological substrates [3, 42]. Apathy is more common in patients with right than left hemisphere damage [14, 43]. Neuroimaging studies showed that apathy was correlated with decreased right temporoparietal perfusion in Alzheimer's patients [44] and with decreased gray matter volume in the right anterior cingulate in older adults [45]. In PD, apathy was correlated with low gray matter density in bilateral inferior frontal and inferior parietal gyrus, right cingulate and right precuneus in one study [46] and with low volume of the right medial temporal lobe in another study [12].

Anxiety is also a very common and yet under studied nonmotor symptom of PD. Prevalence of anxiety disorders in PD varies, with estimates up to 49% [18, 47–49]. Previous studies documented a negative impact of anxiety symptoms on severity of PD [49], subjective motor symptoms [50] and on health-related quality of life in PD [51, 52]. Anxiety also may be associated with depression in PD. Some authors reported overlapping symptoms of depression and anxiety in PD patients [49]. However, dissociation of anxiety and depression in PD was also documented by several studies [53]. Previous studies provide support to the notion that anxiety and depression refer to different neural mechanisms in patients with PD (reviewed in [53]). Earlier studies reported anxiety associated with various cognitive deficits [54–56], though few studies have investigated the effect of anxiety on cognition in PD. One study found that PD patients with symptoms of anxiety demonstrated poorer performance of various measures of executive functioning, attention, processing speed, and episodic memory [57]. These findings remained significant after symptoms of depression were controlled. Ryder and colleagues also reported a negative association between anxiety and cognitive performance in PD [58]. Anxiety is a common feature of left hemisphere involvement [59–61]. Lesion studies showed that individuals with left hemisphere lesions may be particularly at risk of developing anxiety after stroke [62]. Anxiety was also associated with greater left-hemisphere activation in healthy adults [63]. More recently, anxiety was associated with bilateral middle frontal, anterior cingulate, and left parahippocampal/amygdala region in healthy adults [64]. Another study of healthy adults that reported left amygdala volumes significantly predicted anxiety scores and found an overlap between areas associated with both amygdala volume and anxiety scores in the orbitofrontal cortex and the left inferior parietal region [65]. Apathy and anxiety both have a very significant effect on quality of life and substantially increase patient disability [52, 66, 67]. Understanding their effect on cognition, elucidating underlying neural mechanisms and evaluating the implications for management of these neuropsychiatric symptoms PD are urgently needed.

This study aimed to further elucidate the phenomenology of neuropsychiatric symptoms in PD by (1) assessing the effect of neuropsychiatric status on cognitive function, in particular, the relation of apathy, anxiety, and cognition; and (2) relating them to the hemispheric asymmetry of initial presentation (side of onset of motor symptoms). Previous neuroimaging studies demonstrated that different neuropsychiatric conditions are differentially mediated by lateralized brain areas (as outlined above), which led us to expect that the expression of apathy and anxiety in PD would be lateralized as well. We hypothesized that PD patients would show different neuropsychiatric and neurocognitive profiles depending on the side of disease onset, with LPD exhibiting cognitive deficits predominantly in the nonverbal domain, which would be related to apathy, and RPD showing deficits predominantly in verbal domain, which would be related to anxiety. Apathy and anxiety are important determinants of quality of life in PD. Early detection and treatment of both conditions may enhance patients' everyday functioning and thus protect their quality of life.

## 2. Methods

### 2.1. Participants

The participants included 22 nondemented individuals with PD and 22 normal control adults (NC), who were matched on sociodemographic variables (see Table 1). All participants scored 28 or higher on the Mini-Mental State Examination [68] and were not demented. Participants with PD were recruited from the Parkinson Clinic of the Department of Neurology, Boston Medical Center, and through local support groups. Healthy age-matched control participants (NC) were recruited for the study from the community. The research was approved by Boston University's Institutional Review Board. Participants were required to be native speakers of English. Exclusion criteria included coexisting cancer, serious cardiac disease, other serious chronic medical illness, prior intracranial surgery, history of traumatic brain injury, psychiatric (not including diagnosis of depression or anxiety) or neurological diagnoses other than PD, history of alcoholism or other drug abuse, history of eye disease or other visual abnormalities, and use of psychoactive medications, except for use of antidepressants and anxiolytics in the PD group, which are commonly prescribed. PD clinical staging was determined by their neurologist based on the widely used index of motor disability, Hoehn and Yahr scale [69]. All PD participants were stages II-III (mild to moderate bilateral). The average duration of disease was 8.7 years (SD = 3.8). PD diagnosis was made by patients' neurologists, using UK Parkinson's Disease Society Brain Bank clinical diagnostic criteria [70]. Side of motor symptom onset was obtained by patient report as well as from the patient's neurologist records. Eleven patients had right body side onset of motor symptoms (RPD: 6 men, 5 women), and 11 had left-side onset (LPD: 5 men, 6 women). RPD and LPD groups did not differ in age, education, mental status, Hoehn and Yahr stage, or disease duration. There were no group differences between male and female PD participants in age, education, mental status, Hoehn and Yahr stage, or disease duration. All PD participants received daily dopamine replacement therapy and/or dopamine receptor agonists. Levodopa equivalent dosages (LED) were calculated based on previous reports with LED: (regular levodopa dose × 1) + (levodopa controlled-release dose × 0.75) + (pramipexole dose × 67.0) + (ropinirole dose × 16.67) + (rotigotine × 16.67) + (pergolide dose and cabergoline dose × 67.0) + (bromocriptine dose × 10) + ([regular levodopa dose + levodopa controlled-release dose × 0.75] × 0.25) if taking tolcapone or entacapone [71]. There were no significant differences in RPD versus LPD patient groups in levodopa dose equivalents and dopamine agonists. None of the PD participants were taking anticholinergic medications, and three were taking some form of sleep medication. PD participants were tested while being administered their antiparkinsonian medications (in their “on state”).

### 2.2. Procedure

Before participating, each individual provided informed consent in accordance with regulations of the Boston University Institutional Review Board. All participants were administered standardized measures of neuropsychiatric functioning, and a series of neuropsychological measures sensitive to PD-associated cognitive impairments. Cognitive tests were chosen to assess a range of cognitive abilities in verbal and nonverbal domains, to specify the role of neuropsychiatric symptoms in cognitive functioning in nondemented individuals with PD. All tests were administered and scored according to standard procedures. Because the PD and NC groups were matched on age, education, and male : female ratio, we compared and reported raw scores for all tests.

#### 2.2.1. Neuropsychiatric Status Assessment

We assessed anxiety and apathy using standardized self-report measures.

Apathy was assessed using the modified 14-item *Apathy Evaluation Scale *(AES) [11, 72]. Items are rated on a 0-to-3 Likert scale. Scores range from 0 to 42, with higher scores indicative of more severe apathy level. Sample items include, “Are you interested in learning new things?” and “Are you indifferent to things?” AES and its modified version were reported to have excellent psychometric properties and have been used in studies of Parkinson's disease [11, 13] and other frontostriatal disorders [3, 36, 73]. Total score was the dependent measure.

*The Beck Anxiety Inventory* (BAI) [74] is a 21-item self-report instrument that assesses the existence and severity of symptoms of anxiety. There is a four-point scale for each item ranging from 0 to 3. Total score in the range of 0–13 is considered indicative of minimal or no depression, 14–19 is mild, 20–28 is moderate, and 29–63 is severe [75]. The BAI was reported to differentiate well between anxious and nonanxious groups in a variety of clinical settings. 

*The Beck Depression Inventory, Second Edition* (BDI-II) [76], is a 21-item self-report instrument that assesses the existence and severity of symptoms of depression as listed in the American Psychiatric Association's Diagnostic and Statistical Manual of Mental Disorders, Fourth Edition (DSM-IV) [77]. There is a four-point scale for each item ranging from 0 to 3. Total score in the range of 0–13 is considered indicative of minimal or no depression, 14–19 is mild, 20–28 is moderate, and 29–63 is severe.

#### 2.2.2. Cognitive Functioning Assessment

The neuropsychological series included a number of tests that we expected to be sensitive to frontostriatal and parietal dysfunction (attention, executive function, visuospatial ability) as well as tests that we expected would elicit relatively unimpaired performance in the nondemented PD group (naming abilities and memory). The focus of interest was also on the dissociation between the performance on the verbally and nonverbally mediated tasks, which would be most relevant for the association between the side of onset and cognitive functioning in nondemented persons with PD.

**Verbal Domain**

- *Controlled Oral Word Association Test* [78]. This is a standardized test of verbal (phonemic) fluency, in which participants were required to generate words beginning with a particular letter (F, A, S). Total number correct within a 60-s time period for each condition was recorded.
- *Digit Span*, Wechsler Memory Scale III [79], is a standardized measure of efficiency of attention (Forward Span) and working memory (Backward Span) in verbal domain [61]. The standard total score was used for the group comparison.
- *Clock Reading Test* [27]. The participants are asked to identify and “read” the time shown on each of the 10 clocks presented on a standard paper. Total number of correct responses and time to completion is recorded.
- *The Boston Naming Test *(BNT) [80] is a test of confrontation naming, in which the participant names 60 black and white line drawings of objects presented one at a time. The total number correct was recorded.
- *California Verbal Learning Test-II* (CVLT-II) [81] is a test of list-learning verbal memory. Immediate Recall, Delayed Recall, and number of errors were recorded.

**Nonverbal Domain**

- *Raven's Coloured Progressive Matrices* (RCPM) [82]. This is a standardized measure assessing visuospatial skills and reasoning ability. The task is to choose one of six possible completions of an incomplete pattern matrix. Total score (the number correct out of 36 items) was recorded.
- *The Trail Making Test* (TMT) [83] is a standardized test of psychomotor speed and executive functioning. Trails B subtest is a measure of combined visual search, psychomotor speed, and cognitive flexibility, assessing the ability to shift and maintain the response set, where participants sequentially alternate between alpha-numeric sequences (1-A-2-B, etc.). Time to completion was used for the group comparisons.
- *Spatial Span*, Wechsler Memory Scale III [79], is standardized measure of efficiency of attention and working memory (Backward Span) in nonverbal domain [61]. The standard total score was used for the group comparison.
- *Visual Symbol Search Test* (VS) [84] provides a measure of visual scanning abilities and sustained attention. Participants search and cancel the target symbol in the nonverbal array. Time to completion was used for the group comparison.
- *Rey-Osterrieth Complex Figure* Test (ROCF) [85]. In an assessment of visuospatial memory, participants were asked to recall the abstract figure image by redrawing it immediately after copying (incidental) and again after 25 minutes (delayed recall). We employed the 36-point scoring system evaluating the presence and accuracy of the 18 elements of the ROCF, and a total score was recorded.

#### 2.2.3. Statistical Analyses

To analyze differences between PD and NC groups, independent samples *t*-tests (2-tailed) were used. Pearson correlations were performed to examine associations between apathy and anxiety ratings and neuropsychological performance within the PD and NC groups separately. Multiple regression analyses were performed to examine the relative contribution of neuropsychiatric and disease-related variables to cognitive functioning in PD, using apathy and anxiety total scores and disease duration as predictors, and neuropsychological test scores as criterion variables. Analyses of performance of men and women in both the PD and NC groups revealed no significant differences in neuropsychological profile or mood ratings, and data were accordingly collapsed across gender.

## 3. Results

This study used a within-subject design, with each participant receiving all assessments. The results are divided into two sections: the first section presents findings regarding the effects of PD on neuropsychiatric status and cognitive function; the second section relates neuropsychiatric status to cognitive performance, and more specifically to the hemispheric asymmetry of initial presentation (side of onset of motor symptoms).

### 3.1. Effect of PD on Neuropsychiatric Status and Cognitive Function

**Apathy**

Independent groups *t*-tests revealed that the PD participants reported significantly more apathy symptoms than the NC group (*F*(1,43) = 0.53, *P* < 0.04). AES mean total score was 8.4 (SD = 3.9) for PD and 6.3 (SD = 2.9) for NC. We also conducted RPD and LPD subgroup comparisons that revealed significant differences between the two PD subgroups (*F*(1,21) = 0.11, *P* < 0.02), with RPD mean total AES score = 10.42 (SD = 3.3) and LPD score = 6.9 (SD = 3.8). Apathy ratings (AES total score) significantly correlated with disease duration (years since onset) (*r* = 0.57, *P* < 0.016).

**Anxiety**

The PD group reported significantly more anxiety symptoms than the NC group (*F*(1,43) = 1.49, *P* < 0.03). BAI mean total score was 9.3 (SD = 8.1) for PD and 4.9 (SD = 5.2) for NC. There was a significant association between anxiety (BAI total score) and disease duration (years since onset) (*r* = 0.58, *P* < 0.015). PD subgroup comparisons did not show significant difference between RPD and LPD on BAI (*P* > 0.46).

**Cognitive Performance**

To examine whether the PD group exhibited cognitive deficits compared to the NC group, we conducted independent groups *t*-tests across neuropsychological assessments. The PD group performed more poorly than the NC group on a number of tests. Significant group differences were observed on verbally and nonverbally mediated measures of executive and visuospatial functioning (see Table 2).

### 3.2. Relation between Neurocognitive and Neuropsychiatric Status and Side of Onset

Further analyses revealed correlations between neuropsychiatric (AES and BAI) and neuropsychological measures (Table 3). The cognitive differences remained significant when controlling for apathy (all *P*s < 0.042) and for anxiety (all *P*s < 0.035). As predicted, we observed a different pattern of performance by the two PD subgroups. In LPD, apathy but not anxiety was associated with performance on *non-verbally* mediated executive function and visuospatial measures [TMT B, Spatial Span, and Visual Search], whereas in RPD, anxiety but not apathy significantly correlated with performance on *verbally* mediated tasks [BNT, CVLT, Clock Reading, and FAS] (Table 3).

## 4. Discussion

We examined the effect of PD on neuropsychiatric status and its relation to side of onset of motor symptoms and cognition in nondemented individuals with PD. First, PD patients reported significantly more apathy and anxiety symptoms than the NC group. There were also PD-related changes in multiple cognitive domains. The affected domains included attention, executive function, and visuospatial function, which is reflective of frontostriatal and parietal dysfunction associated with PD. Second, the extent of apathy and anxiety significantly correlated with performance on neuropsychological measures of attention, executive, and visuospatial function. Third, we observed different neuropsychiatric and neurocognitive profiles depending on the side of disease onset, in support of our hypothesis. Specifically, we had predicted that patients with LPD would exhibit cognitive deficits predominantly in the visuospatial domain, which would be related to apathy. In contrast, RPD would show cognitive deficits in verbal domain, which would be associated with anxiety symptoms.

To our knowledge, this is the first study to demonstrate a differential association of apathy and anxiety to cognitive dysfunction in PD, in relation to the side of onset of motor symptoms.

Apathy and anxiety are common clinical features of PD, and both are important predictors of everyday functioning and quality of life. Previous neuroimaging and neuropsychological studies related these neuropsychiatric conditions to PD-associated dysfunction. However, the role of apathy and anxiety in cognitive dysfunction and their underlying neurophysiological substrates remain a subject of ongoing investigation. Several studies related apathy to cognitive dysfunction in PD and other frontostriatal disorders (reviewed earlier in this paper). While anxiety was also associated with various cognitive deficits [54–56], there have been only a few studies that investigated the effect of anxiety on cognition in PD [58]. We assessed the effect of both apathy and anxiety on cognitive function in PD; in particular, the relation of apathy and anxiety to lateralized cognitive domains (verbal versus visuospatial).

Consistent with previous literature, our PD patients reported significantly more apathy and anxiety symptoms than the NC group, implicating disruption of specific frontostriatal neural pathways. Also, as expected, we observed PD-related changes in multiple cognitive domains in our sample of nondemented patients, including attention, executive function, and visuospatial function. These findings provide additional support to earlier work, which suggested that the higher prevalence of apathy and anxiety in PD, as compared to general population, reflects PD-related dysfunction of frontostriatal systems.

As suggested by previous research (reviewed above), various neuropsychiatric conditions are differentially mediated by lateralized brain areas, which led us to hypothesize that the expression of apathy and anxiety in PD also would be lateralized. Specifically, we expected that PD patients would show different neuropsychiatric and neurocognitive profiles depending on the initial side of motor onset. We predicted that LPD patients would exhibit cognitive deficits predominantly in the visuospatial domain, which would be related to apathy. We also examined the potential mediating influence of anxiety on cognitive performance in PD. Specifically, we expected that RPD patients would show cognitive deficits predominantly in the verbal domain, which would be related to anxiety.

As predicted, we observed different neuropsychiatric and neurocognitive profiles in our PD subgroups. Apathy and anxiety differentially correlated with performance on neuropsychological measures. The lateralized cognitive domain (verbal versus visuospatial) was the mediating factor in this paradigm. The observed dissociation between apathy and anxiety and distinct cognitive domains suggested anatomically and functionally distinct neural substrates. Consistent with our hypotheses, in LPD, apathy but not anxiety was associated with performance on nonverbally mediated executive function and visuospatial tasks. This finding is consistent with earlier reports of apathy being related to right-hemisphere dysfunction [12, 14, 43, 46]. In RPD, by contrast, anxiety but not apathy significantly correlated with performance on verbally mediated tasks. This finding provides additional support to lesion studies and neuroimaging reports relating anxiety to left-hemisphere dysfunction [59–61, 63, 65]. Taken together, our results demonstrated a differential association of apathy and anxiety with cognition in PD.

In this study, we observed no significant differences between the RPD and LPD subgroups in anxiety ratings, and higher levels of apathy in the RPD group as compared to LPD. The lack of group differences in anxiety level and higher rate of apathy in the RPD group may potentially reflect variations in the degree of lateralized pathology within each subgroup in our nondemented PD sample. Future investigations with larger patient samples and a wider range of disease duration and disease characteristics may shed light on changes in the lateralization and expression of neuropsychiatric deficits across time, as PD-associated neuropathological changes may become less lateralized with disease progression. Longitudinal studies are needed to address the effect of the treatment of apathy and anxiety on cognitive function and quality of life in PD.

Although limited by the size of our study sample, our results nevertheless indicate a significant relation between neuropsychiatric status and disease duration in nondemented PD. We found that apathy and anxiety ratings were associated with PD duration. The identification and treatment of these neuropsychiatric conditions in patients with PD is very important, as both apathy and anxiety have significant impact on quality of life and substantially increase patient disability [66, 67]. Apathy and anxiety are treatable conditions, and timely screening and intervention may protect the quality of life and reduce disability in individuals with PD.

In conclusion, this study examined the association between neuropsychiatric symptoms and cognitive function in non-demented individuals with PD. Our findings supported the notion that the higher rate of apathy and anxiety in PD than in the general population may reflect a direct consequence of the damage to the frontostriatal system and its cortical connections, resulting in both neuropsychiatric and neurocognitive deficits. Examination of the relation between apathy and anxiety and distinct cognitive domains suggested anatomically and functionally distinct neural substrates. The observed dissociation between RPD and LPD neuropsychiatric status and cognitive performance also points to distinct underlying neural substrates within corticostriatal-thalamocortical circuits. These results indicate specific relations of apathy and anxiety to cognition in PD. Finally, we found that apathy and anxiety ratings were associated with disease duration. The results of this study stress the importance of identifying and treating these neuropsychiatric conditions in PD patients.

## Figures and Tables

**Table 1:** Demographic and clinical variables in PD and NC participants. Means (SD) are reported unless otherwise indicated.

	NC	RPD	LPD
*N*	22	11	11
Age	61.3 (6.9)	63.2 (6.0)	61.3 (6.2)
Years of Education	16.0 (1.9)	15.5 (2.2)	15.9 (3.0)
M : F	11 : 11	6 : 5	5 : 6
MMSE (total)	29.8 (0.5)	29.1 (0.9)	29.5 (0.8)
BDI-II (total)	4.6 (4.1)*	7.3 (3.5)	7.0 (4.0)
Disease Duration (Years)	n/a	8.4 (3.7)	9.0 (4.0)
Hoehn and Yahr stage^*∧*^	n/a	2 (2-3)	2 (2-3)
LED	n/a	475.2 (294.4)	497.5 (329.7)

BDI-II: Beck Depression Inventory-II.

* Significantly different from PD groups (*P*s < 0.04).

^*∧*^Median (range).

n/a: not applicable.

LED: levodopa equivalent dosage.

**Table 2:** Neuropsychological performance; raw score mean values (SD).

	NC	RPD	LPD
Verbal Domain			
FAS total	50.2 (16.3)	45.2 (14.4)	41.5 (7.5)*
Digit span forward total	12.1 (2.2)	10.6 (1.9)	11.2 (1.8)
Digit span backward total	8.8 (2.6)	6.6 (2.8)*	6.8 (1.6)**
Clock reading total	48.5 (18.8)	64.2 (15.3)*	62.5 (24.1)
BNT total correct	56.4 (3.2)	57.3 (3.5)	55.9 (5.1)
CVLT-II immediate recall total	11.4 (2.2)	11.7 (3.5)	11.3 (3.2)
CVLT-II delayed recall total	10.8 (2.8)	11.1 (3.5)	8.9 (3.7)

Nonverbal Domain			
RCPM total	34.1 (2.1)	31.1 (4.3)**	31.8 (3.0)*
TMT B time	66.8 (31.4)	86.8 (40.3)	99.3 (90.9)
Spatial span forward total	8.4 (1.7)	7.5 (1.9)	8.0 (1.1)
Spatial span backward total	7.8 (1.8)	7.2 (1.2)	7.3 (2.2)
VS search time	82.2 (21.7)	130.9 (45.2)**	137.2 (93.7)**
ROCF immediate recall total	17.1 (6.5)	15.6 (7.3)	17.8 (7.0)
ROCF delayed recall total	17.9 (6.1)	15.3 (8.3)	17.0 (7.2)

Significantly different from NC group: *P < 0.03, **P < 0.007.

FAS: Controlled Oral Word Association Test (F, A, S),

BNT: Boston Naming Test,

CVLT-II: California Verbal Learning Test-II,

RCPM: Raven's Coloured Progressive Matrices,

ROCF: Rey-Osterrieth Complex Figure,

VS: Visual Symbol Search.

**Table 3:** Correlations between RPD and LPD neuropsychological performance and apathy (AES) and anxiety (BAI) ratings.

	Apathy	Anxiety
Verbal domain		

RPD		
FAS total correct	ns	−0.74*
Clock reading total errors	ns	0.84**
Clock reading impulsive errors	ns	0.89**
BNT total correct	ns	−0.84**
CVLT errors (intrusions)	ns	0.89**

Nonverbal domain		

LPD		
TMT B time	0.73*	ns
TMT B errors	0.64*	ns
Spatial span forward total	−0.68*	ns
Spatial span backward total	−0.69*	ns
VS search time	0.65*	ns
VS search errors (omissions)	0.70*	ns

*P < 0.025; **P < 0.005.

TMT: Trail Making Test,

VS: Visual Symbol Search.
